# *RARS2* mutations in a sibship with infantile spasms

**DOI:** 10.1111/epi.13358

**Published:** 2016-04-08

**Authors:** Adeline Ngoh, Jose Bras, Rita Guerreiro, Esther Meyer, Amy McTague, Eleanor Dawson, Kshitij Mankad, Roxana Gunny, Peter Clayton, Philippa B. Mills, Rachel Thornton, Ming Lai, Robert Forsyth, Manju A. Kurian

**Affiliations:** ^1^ Molecular Neurosciences, Developmental Neurosciences ProgrammeUCL‐Institute of Child HealthLondonUnited Kingdom; ^2^ Department of NeurologyGreat Ormond Street Hospital for Children NHS Foundation TrustLondonUnited Kingdom; ^3^ Department of Molecular NeuroscienceUCL‐Institute of NeurologyLondonUnited Kingdom; ^4^ Laboratory of NeurogeneticsNational Institute on AgingNational Institutes of HealthBethesdaMarylandU.S.A; ^5^ Department of Paediatric NeurologyGreat North Children's HospitalRoyal Victoria InfirmaryNewcastle upon TyneUnited Kingdom; ^6^ Department of RadiologyGreat Ormond Street Hospital for Children NHS Foundation TrustLondonUnited Kingdom; ^7^ Genetics and Genomic Medicine ProgrammeUCL‐Institute of Child HealthLondonUnited Kingdom; ^8^ Metabolic Medicine UnitGreat Ormond Street Hospital for Children NHS Foundation TrustLondonUnited Kingdom; ^9^ Department of NeurophysiologyGreat Ormond Street Hospital for Children NHS Foundation TrustLondonUnited Kingdom; ^10^ Department of NeurophysiologyRoyal Victoria InfirmaryNewcastle upon TyneUnited Kingdom; ^11^ Institute of NeuroscienceNewcastle UniversityNewcastle upon TyneUnited Kingdom

**Keywords:** *RARS2*, Infantile spasms, Whole exome sequencing, Pontocerebellar hypoplasia type 6

## Abstract

Pontocerebellar hypoplasia is a group of heterogeneous neurodevelopmental disorders characterized by reduced volume of the brainstem and cerebellum. We report two male siblings who presented with early infantile clonic seizures, and then developed infantile spasms associated with prominent isolated cerebellar hypoplasia/atrophy on magnetic resonance imaging (MRI). Using whole exome sequencing techniques, both were found to be compound heterozygotes for one previously reported and one novel mutation in the gene encoding mitochondrial arginyl‐tRNA synthetase 2 (*RARS2*). Mutations in this gene have been classically described in pontocerebellar hypoplasia type six (PCH6), a phenotype characterized by early (often intractable) seizures, profound developmental delay, and progressive pontocerebellar atrophy. The electroclinical spectrum of PCH6 is broad and includes a number of seizure types: myoclonic, generalized tonic–clonic, and focal clonic seizures. Our report expands the characterization of the PCH6 disease spectrum and presents infantile spasms as an associated electroclinical phenotype.

The early infantile epileptic encephalopathies (EIEEs) comprise a heterogeneous group of disorders characterized by early onset seizures, developmental delay/regression, abnormalities on electroencephalography (EEG), and often poor neurologic outcome.^1^ A number of electroclinical syndromes are described, based on age of onset, seizure type, and electrographic findings. Pontocerebellar hypoplasia (PCH) is an emerging clinical entity describing a group of heterogeneous severe developmental disorders affecting growth and function of the brainstem and cerebellum. At least 10 forms of PCH are recognized, and many subtypes have an EIEE phenotype, with different seizure semiology.^2^

We describe a family of two affected boys with pontocerebellar hypoplasia type 6 and mutations in *RARS2,* presenting with infantile spasms. In this report, we delineate their clinical phenotype, neuroradiologic features, and molecular genetic findings.

## Methods

### Acquisition of clinical cases

Following extensive clinical workup, the reported family was recruited for research molecular genetics investigations. Informed written consent was obtained for all participants. The study was conducted in keeping with the Declaration of Helsinki.

### Molecular genetics

Whole exome sequencing was performed in both affected individuals. DNA samples were captured using Illumina's TruSeq and sequenced on Illumina's HiSeq2000 using 100 base pair, paired‐end reads. We generated 11,845,539,100 and 11,047,647,300 total bases, which leads to mean on‐target coverage of 87.1× and 82.4× per sample. Data were analyzed according to Genome Analysis Tool Kit's Best Practices.^3^, ^4^ Briefly, this consisted of flagging duplicate reads, realignment around indels, base recalibration, and variant calling on both samples simultaneously using the Haplotype Caller tool. Variant qualities were then recalibrated according to Best Practices recommendations for exomes. Variants that fulfilled the following criteria were considered for follow‐up analyses: (1) variants with a minor allele frequency < 0.01 in established publicly available databases (1000 Genomes, ESP6500, and ExAC) and in ~1,800 in‐house analyzed exomes; (2) compound heterozygous changes consistent with autosomal recessive inheritance; (3) shared variants on the X chromosome, consistent with X‐linked inheritance; and (4) prediction of putative pathogenicity based on mutation type, or in silico prediction of effects on protein function and/or structure, using Polyphen (http://genetics.bwh.harvard.edu/pph2/). The identified *RARS2* sequence variations were confirmed by direct Sanger sequencing using standard methods.

## Results

### Clinical cases

We describe two affected male siblings born to nonconsanguineous Caucasian British parents. Family history was unremarkable.

*Patient 1* was delivered at term with a birthweight of 3.57 kg (50th–75th percentile), and head circumference of 35 cm (50th percentile). He presented at 40 h of age with unexplained hypoglycemia (blood glucose <0.1 mmol/L), which normalized on feeding. At 5 weeks of age, he manifested clonic seizures, which responded to phenobarbitone. At 6 months of age, he developed infantile spasms. This responded partially to high‐dose vigabatrin with an approximate 50% reduction in spasms. From the age of 11 months, he developed increasingly pharmacoresistant epilepsy, with tonic and generalized tonic–clonic seizures, refractory to trial of several anticonvulsants, including sodium valproate, topiramate, levetiracetam, phenytoin, phenobarbitone, clonazepam, and vigabatrin. He required multiple admissions to the intensive care unit for prolonged seizures. His parents reported a possible response to pyridoxine therapy, although this was neither dramatic nor sustained, and therefore not suggestive of an inborn error of metabolism affecting pyridoxal phosphate homeostasis. Introduction of the ketogenic diet at 2.5 years resulted in a modest reduction in seizure burden.

He developed a social smile at 5 weeks, but did not achieve any further developmental milestones. Visual impairment was apparent clinically by 5 months of age. Due to progressive bulbar dysfunction, nasogastric feeding was initiated at 3 years. There was progressive decline in his head circumference from the 50th percentile at birth, to the 25th percentile at 22 weeks, to below the 0.4th percentile at his last clinical examination. Currently at the age of 11 years, he has severe neurodisability with peripheral limb spasticity, scoliosis, and significant seizure burden.

**Figure 1:**
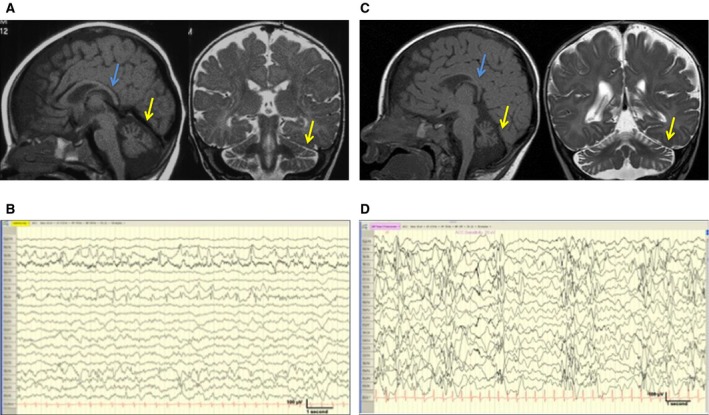
Radiologic and electrographic features. (**A**) MRI brain scan for patient 1, 7 months of age: Sagittal T_1_‐weighted and coronal T_2_‐weighted imaging showing generalized reduction in white matter bulk and marked cerebellar atrophy (yellow arrow) and corpus callosum thinning (blue arrow) with relative sparing of the pons. (**B**) Last EEG of patient 1, at 6 years of age, continued to show frequent epileptiform activity more prevalent over the posterior regions. (**C**) MRI brain scan of patient 2, age 6 months: Sagittal T_1_‐weighted and coronal T_2_‐weighted imaging shows marked cerebellar atrophy (yellow arrow), thinning of the corpus callosum (blue arrow), relative sparing of the pons, and reduction in white matter bulk. (**D**) EEG of patient 2, age 5 months: EEG showed frequent bursts of polymorphic high‐amplitude focal sharp delta/theta over both posterior temporal regions with a bias to the right.

Magnetic resonance imaging (MRI) at 7 months showed a generalized lack of white matter bulk with a thin corpus callosum, and a small cerebellum, with relative sparing of the pons (Fig. 1A). EEG studies at 5–6 weeks of age were normal. At the age of 6–8 months, the EEG was disorganized and asynchronous in some parts with frequent bi‐hemispheric generalized bursts of epileptiform activity. This showed features of a prehypsarrhythmia phase,^5^ but did not meet criteria for hypsarrhythmia. Since the age of 11 months, EEG studies have generally shown asymmetrical frequent polyrhythmic epileptiform activity maximal in the posterior regions, and right hemisphere (Fig. 1B). Neurometabolic investigations were unremarkable, with the exception of an intermittently raised serum lactate (2.79–4.85 mmol/L, normal range 0.70–2.10 mmol/L) early in the disease course. Cerebrospinal fluid (CSF) lactate and pyruvate levels were within normal limits. CSF and plasma pyridoxal phosphate levels were normal, as was pyridoxamine 5′‐phosphate oxidase (*PNPO*) mutation analysis. Urine organic acid analysis at 7 months (but not at other times) showed increased excretion of lactate and citric acid cycle intermediates. Results of other neurometabolic investigations were within normal limits. A muscle biopsy carried out at 1 year of age showed normal histology, electron microscopy, and mitochondrial respiratory chain enzyme activity (complex I–IV).

*Patient 2* is the younger brother of the index patient. Perinatal history was unremarkable. He presented at 8 weeks of age with clonic seizures, which responded to phenobarbitone. At 5 months, he too developed infantile spasms, which did not respond to biotin, folinic acid, or pyridoxal phosphate, but improved on vigabatrin, with an approximate 50% reduction in seizure frequency. By 16 months, he developed refractory epilepsy with multiple seizure types, including myoclonus and tonic spasms. A second trial of pyridoxal phosphate undertaken at this time reportedly improved energy levels but did not reduce seizure frequency.

Developmentally, his visual behavior was abnormal from 8 weeks of age. Over the years, he developed axial hypotonia and four‐limb spasticity. Acquired microcephaly was also evident. His head circumference fell from the 50th percentile at birth to the 2nd percentile by 6 years of age. Full gastrostomy feeds were required by 3 years of age. Currently, at the age of 6 years, he continues to have daily seizures despite multiple anticonvulsants and ketogenic diet.

A brain MRI scan (6 months), showed findings similar to his brother's (Fig. 1C). EEG at 5 months of age showed frequent bursts of polymorphic high‐amplitude focal sharp delta/theta over both posterior temporal regions with a bias to the right (Fig. 1D). This evolved to show activity similar to his brother's with periods of asynchrony. At 2.5 years of age, EEG continued to show frequent epileptiform activity over the posterior temporal and occipital regions. Neonatal serum lactate levels are not available. A recent serum lactate level at the age of 6 years was normal (1.6 mmol/L). CSF lactate and pyruvate levels were normal. CSF pyridoxal phosphate level was at the lower end of the normal range at 16 nmol/L (normal 14–92 nmol/L). A simultaneous plasma pyridoxal phosphate was normal at 46 nmol/L (15–73 nmol/L). Urine alpha aminoadipic semialdehyde was also normal. CSF glycine was mildly elevated. All other neurometabolic investigations were normal. He did not have a muscle biopsy. Clinical microarray testing revealed no significant pathogenic copy number variants.

### Molecular genetic investigation

Two heterozygous variants were identified in *RARS2*, independently confirmed by Sanger sequencing and showed appropriate familial segregation: (1) a 3 bp deletion in exon 7 (NM_020320: c.472_474del, p.K158del; 6:88,239,290) and (2) a novel missense mutation in exon 10 (NM_020320: c.848T>A, p.L283Q; 6:88,255,394). The 3 bp deletion has only been reported in three European Americans in the heterozygous state in the National Heart Lung and Blood Institute Exome Sequencing Project (NHLBI ESP) Exome Variant Server and is therefore very rare. The missense variant is absent in the 1000 Genomes database, the NHLBI ESP Exome Variant Server, and dbSNP (v137). PolyPhen‐2 predicts the missense mutation to be likely damaging, with a score of 1.000, targeting the highly conserved amino acid L283. All other possible disease‐causing mutations identified in the two subjects that passed our selection criteria were excluded, leaving *RARS2* as the only candidate gene (Table S1 and Figure S1).

## Discussion

Mutations in *RARS2* have been reported in patients with pontocerebellar hypoplasia type 6 (PCH6, OMIM 611523).^2^, ^6^, ^7^, ^8^, ^9^, ^10^, ^11^, ^12^ The gene encodes the mitochondrial aminoacyl‐tRNA synthetase (ARS) enzyme, arginyl transfer RNA synthetase (MIM 611524). ARSs, such as RARS2, attach amino acids to their cognate tRNA molecules, and play an important role in mRNA translation, a key process in maintaining cell integrity.^13^

Mutations identified in *RARS2‐*PCH, include missense mutations, deletions, and splice‐site variants (Table 1).^2^, ^6^, ^7^, ^8^, ^9^, ^10^, ^11^, ^12^ One *RARS2* mutation in our sibship is novel, and the other previously reported. Glamuzina et al.^8^ report a patient harboring an identical 3 bp deletion (c.472_474del), with the same predicted functional consequences (loss of lysine158). Based on homology with the yeast cytoplasmic arginyl‐tRNA synthetase, K158 is located within the active site domain of the enzyme, and it is postulated that loss of this amino acid may affect catalytic activity.^13^

**Table 1:**
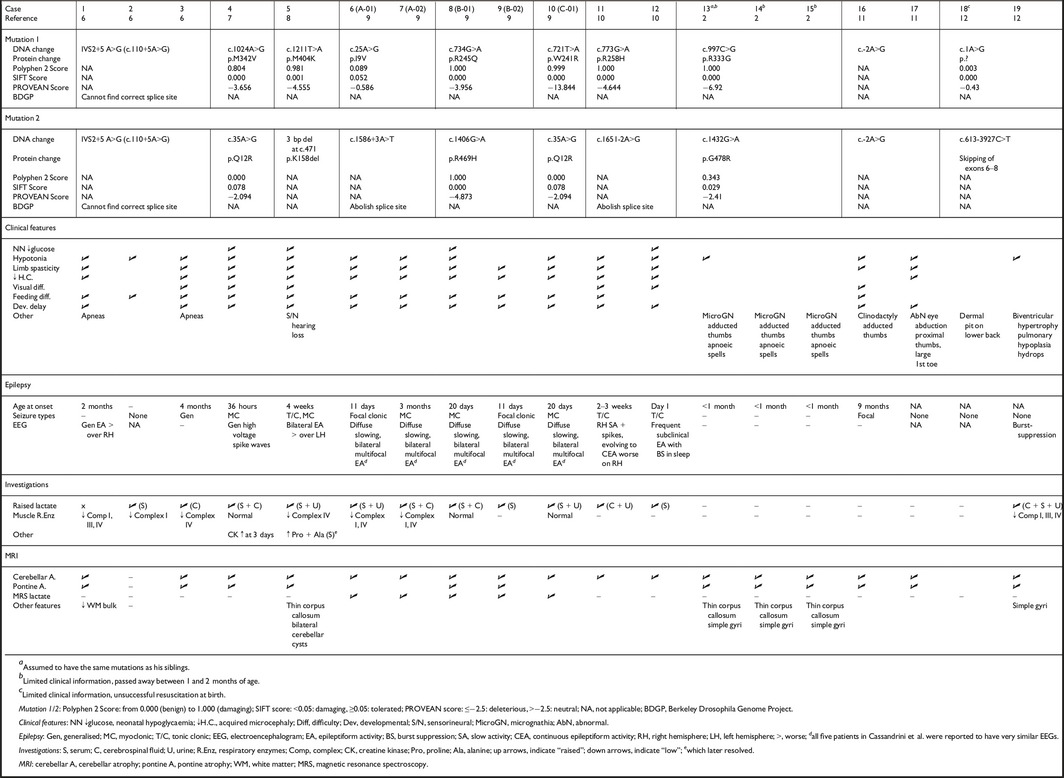
Features of reported patients with mutations in *RARS2*

To date, 19 *RARS2‐*PCH cases from 10 families have been reported (Table 1).^2^, ^6^, ^7^, ^8^, ^9^, ^10^, ^11^, ^12^ Our sibship manifest a number of clinical features common to those reported, including infantile‐onset of disease, pharmacoresistant epileptic encephalopathy, severe neurodevelopmental delay, acquired microcephaly, neonatal hypoglycemia, feeding difficulties, abnormal visual behavior, and early raised serum and/or CSF lactate. Patients with *RARS2* mutations manifest multiple different electroclinical phenotypes including generalized tonic–clonic, focal clonic, and myoclonic episodes.^2^, ^6^, ^7^, ^8^, ^9^, ^10^, ^11^, ^12^ Our patients presented with infantile spasms, which have not been reported in *RARS2* PCH6 previously. However, it is well recognized that the etiology of infantile spasms is heterogeneous (Table S2 and Supporting information references).

From a radiologic perspective, *RARS2*‐PCH is associated with cerebral atrophy and pontocerebellar hypoplasia/atrophy. It is postulated that supra‐and infratentorial atrophy may be progressive over time, and pontine involvement may not be present in the early stages.^6^, ^8^, ^9^, ^10^ In both our patients, MRI, undertaken within the first year of life, revealed a small cerebellum and generalized reduced white matter bulk with an abnormal corpus callosum. Further serial neuroimaging was not possible for medical reasons. Other radiologic features described in reported patients include subdural effusions (secondary to cerebral atrophy) and abnormal myelination. Elevated lactate peak on MR spectroscopy has also been reported in some *RARS2‐*mutation–positive patients;^9^ this investigation was not undertaken in our patients. It is possible that such structural changes can contribute to clinical phenotype. Indeed determinants of the EIEE phenotype are likely to be multifactorial and governed by the underlying gene defect at a cellular level, its effect on neuronal networks within the brain, and any associated brain structural abnormalities.

In conclusion, we report two patients with *RARS2* mutations in the context of severe neurodevelopmental delay, an intractable seizure disorder and abnormal MRI brain scan. Our report expands the *RARS2* mutational spectrum by introducing a novel mutation, and extends the range of electroclinical phenotypes associated with *RARS2‐* PCH6. Our data suggest *RARS2* should be considered in patients with infantile spasms if other PCH features, such as imaging findings, are present. Identification of more *RARS2‐*mutation–positive cases will no doubt provide further insight into the expanding molecular genotype and clinical disease phenotype.

## Disclosure

The authors do not have any conflict of interest to disclose and confirm to have read the Journal's position on issues involved in ethical publication, and affirm that this report is consistent with those guidelines.

## Supporting information


**Figure S1.** Sanger sequence results.

**Table S1.** Exclusion of other possible pathogenic mutations.

**Table S2.** Genetic causes of infantile spasms.
