# Supplementary figures and images for: RARS2 mutations in a sibship with infantile spasms
Source: Epilepsia. 2016 Apr 8;57(5):e97–e102. doi: 10.1111/epi.13358 (PMC4864753; doi:10.1111/epi.13358)

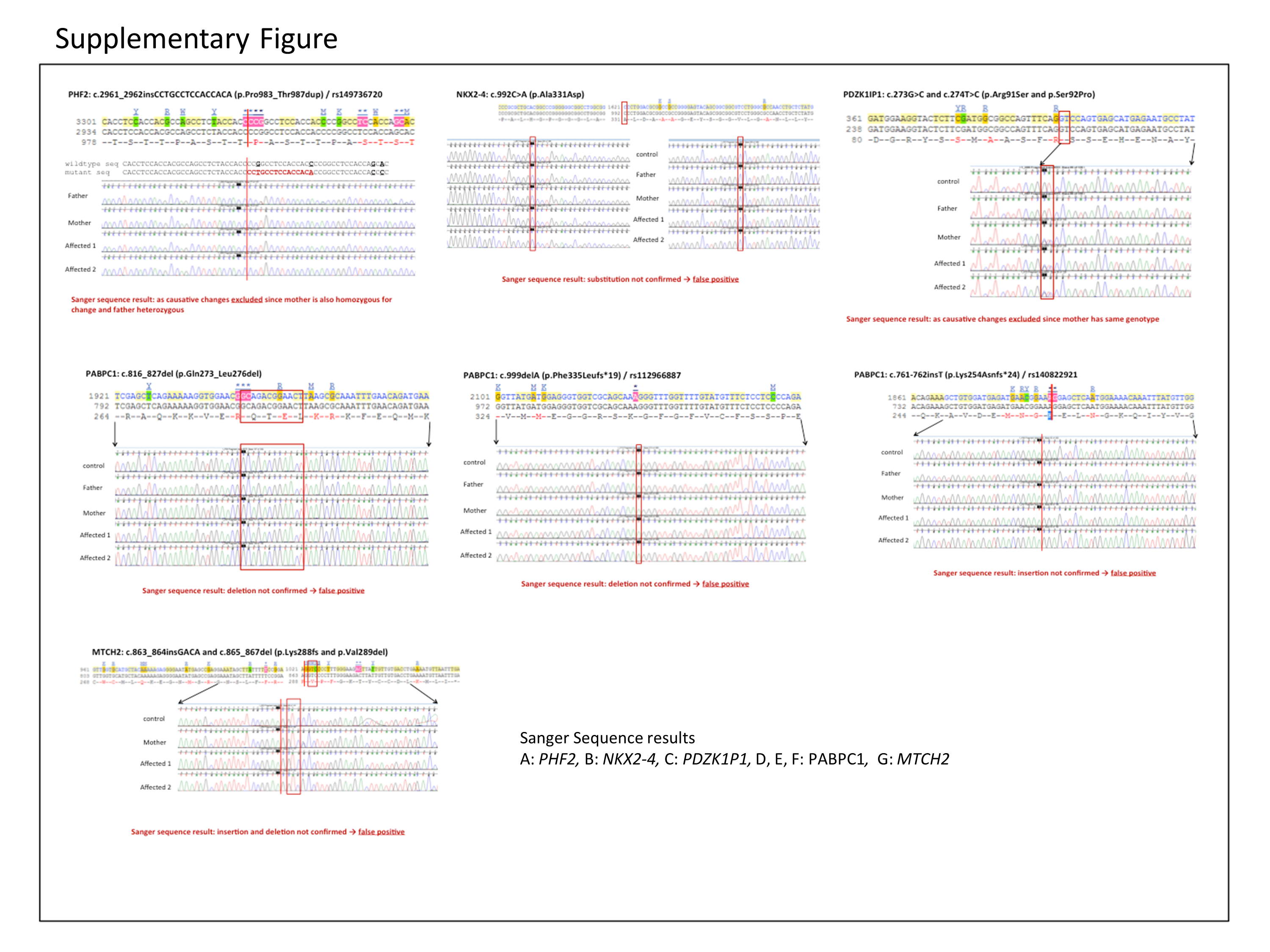

Supplement: Supplementary file 1 — Figure S1. Sanger sequence results. [file EPI-57-e97-s001.tif]
