# Supplementary material for: RARS2 mutations in a sibship with infantile spasms
Source: Epilepsia. 2016 Apr 8;57(5):e97–e102. doi: 10.1111/epi.13358 (PMC4864753; doi:10.1111/epi.13358)
Supplement: Supplementary file 2 — Table S1. Exclusion of other possible pathogenic mutations. [file EPI-57-e97-s002.pdf]

Supplementary Table 1: Exclusion of other possible pathogenic mutations

Gene	Gene reference	DNA change	Protein change	Known rs No.	Polyphen-2	SIFT	PROVEAN	Gene function	Expression	Reason for gene exclusion as a candidate gene
PHF2	NM_005392.3	c.2961_2962ins CCTGCCTCCACC ACA	p.Pro983_Thr987dup<br><br>HGVS: c.2963_2964insTGCCTCCA CCACACC; p.Thr992_Thr996dup	rs149736720	NA	NA	NA	Encodes protein containing zinc finger-like PHD finger; thought to belong to diverse group of transcriptional regulators possibly affecting eukaryotic gene expression by influencing chromatin structure	Widely expressed, including in liver	Mother is homozygous for change
NKX2-4	NM_033176.1	c.992C>A	p.Ala331Asp	Not in 1000 genomes or ESP EVS	Benign Score: 0.244	Tolerated Score: 0.190	Neutral Score: -0.356	Sequence-specific DNA binding transcription factor activity		Not conserved in dog, substitution not confirmed on Sanger sequencing
PDZK1IP1	NM_005764.3	c.274T>C	p.Ser92Pro	Not in 1000 genomes or ESP EVS	Probably damaging Score: 0.997	Damaging Score: 0.014	Deleterious Score: -3.520	PDZK1-interacting protein 1; transmembrane protein	At significant levels only in proximal tubular epithelial cells of kidney; diffusely expressed in various carcinomas originating from kidney, colon, lung and breast	Excluded since mother same genotype as both affected children
		c.273G>C	p.Arg91Ser	Not in 1000 genomes or ESP EVS	Probably damaging Score: 0.989	Damaging; Score: 0.031	Deleterious Score: -4.017			Excluded since mother same genotype as both affected children
PABPC1	NM_002568.3	c.999delA	p.Phe335Leufs*19	rs112966887	NA	NA	NA	Encodes poly(A) binding protein; binds to 3' poly(A) tail of eukaryotic messenger RNAs via RNA-recognition motifs → promoting ribosome recruitment and translation initiation; also required for poly(A) shortening = first step in mRNA decay	Ubiquitous	Not confirmed on Sanger sequencing
		c.816_827del	p.Gln273_Leu276del	Not in 1000 genomes or ESP EVS	NA	NA	NA			Not confirmed on Sanger sequencing
		c.761_762insT	p.Lys254Asnfs*24	rs140822921	NA	NA	NA			Not confirmed on Sanger sequencing
MTCH2	NM_014342.3	c.865_867del	p.Val289del	Not in 1000 genomes or ESP EVS	NA	NA	NA	Mitochondrial carrier 2; substrate transported not known yet; induces mitochondrial depolarization		Not confirmed on Sanger sequencing
		c.863_864insGA CA	p.Lys288fs<br><br>HGV: c.864_865insACAG; p.Val289Thrfs*11	Not in 1000 genomes or ESP EVS	NA	NA	NA			Not confirmed on Sanger sequencing
