# Supplementary material for: RARS2 mutations in a sibship with infantile spasms
Source: Epilepsia. 2016 Apr 8;57(5):e97–e102. doi: 10.1111/epi.13358 (PMC4864753; doi:10.1111/epi.13358)
Supplement: Supplementary file 3 — Table S2. Genetic causes of infantile spasms. [file EPI-57-e97-s003.pdf]

Supplementary Table 2: Genetic causes of infantile spasms

Gene	OMIM gene number	OMIM phenotype number	EIEE designation	Gene function	Mode of inheritance	Phenotypes associated with this gene	References
ARX	300382	308350	EIEE1	Modulates cerebral development through the regulation of differentiation, proliferation and migration of neuronal precursors and cortical interneurones.	X-linked	Infantile spasms/West syndrome<br>X-linked myoclonic seizures<br>Idiopathic infantile epileptic dyskinetic encephalopathy<br>Ohtahara syndrome	Stromme et al 2002[1]<br>Scheffer et al 2002[2]<br>Guerrini et al 2007[3]<br>Kato et al 2007[4], 2010[5]<br>Absoud et al 2010[6]
CDKL5	300203	300672	EIEE2	Not completely understood. May have a role in synaptogenesis	X-linked	Infantile spasms<br>Rett-like phenotype	Kalscheuer et al 2003[7]<br>Weaving et al 2004[8]<br>Scala et al 2005[9]<br>Bahi-Buisson et al 2012[10]<br>Fehr et al 2013[11]
SLC25A22	609302	609304	EIEE3	A glutamate/H+ symporter. Catalyzes either the co-transport of L-glutamate with H+ or its exchange with OH^-	AR Loss of function mutations	Ohtahara syndrome<br>West Syndrome<br>MPSI	Molinari et al 2005[12], 2009[13]<br>Poduri et al 2013[14]
STXBP1	602926	612164	EIEE4	Modulates release of synaptic vesicles/regulates vesicular traffic in neurons	De novo/AD	Ohtahara syndrome Infantile spasms/West Syndrome Non-specific EIEE	Tohyama et al 2008[15]<br>Saitsu et al 2008[16]<br>Deprez et al 2010[17]<br>Otsuka et al 2010[18]
SPTAN1	182810	613477	EIEE5	A cytoskeletal protein that regulates axonal structural stability, receptor binding and actin cross-linking	De novo/AD	Infantile spasms/West Syndrome<br>Non-specific EIEE	Tohyama et al 2008[15]<br>Saitsu et al 2010[19]<br>Hamdan et al 2012[20]<br>Writzl et al 2012[21]<br>Nonoda et al 2013[22]
SCN1A	182389	607208<br>604403<br>609634	EIEE6	Encodes alpha subunit of voltage gated sodium channel essential for generation and propagation of action potentials	De novo/AD	Dravet syndrome/severe myoclonic epilepsy of infancy (EIEE6) GEFS+2<br>Familial febrile seizures 3A<br>Familial hemiplegic migraine 3<br>MPSI<br>Infantile spasms	Claes et al 2001[23], 2003[24]<br>Escayg et al 2000[25]<br>Mantegazza et al 2005[26]<br>Dichgans et al 2005[27]<br>Freilich et al 2011[28]<br>Carranza Rojo et al 2011[29]<br>Wallace et al 2003[30]

Gene	OMIM gene number	OMIM phenotype number	EIEE designation	Gene function	Mode of inheritance	Phenotypes associated with this gene	References
KCNQ2	602235	613720	EIEE7	Encodes a voltage gated potassium channel with an important role in the regulation of neuronal excitability	De novo	Myokymia<br>Benign familial neonatal seizures Early infantile epileptic encephalopathy/ Infantile spasms	Dimassi et al 2015[31]
SCN2A	182390	613721	EIEE11	Encodes one member of the alpha subunit of trans-membrane voltage gated sodium channels, responsible for the generation and propagation of action potentials in neurons and muscles	De novo/AD	Infantile spasms/West syndrome<br>Non- specific EIEE<br>Ohtahara<br>syndrome MPSI	Ogiwara et al 2009[32]<br>Kamiya et al 2004[33]<br>Liao et al 2010[34]<br>Dhamija et al 2013[35]<br>Nakamura et al 2013[36]<br>Hackenberg et al 2014[37]<br>Martin et al 2014[38]<br>Baasch et al 2014[39]
PLCB1	607120	613722	EIEE12	G-protein coupled phospholipase catalyzes the generation of IP3 DAG from IP2 a key step in the intracellular transduction of many extracellular signals. Mutations postulated to disrupt acetlylcholine mediated inhibition leading to excitatory state	AR Loss of function mutations	Infantile Spasms<br>MPSI<br>Non- Specific<br>EIEE	Kurian et al 2010[40]<br>Poduri et al 2011[41]<br>Ngoh et al 2014[42]
ST3GAL3	606494	615006	EIEE15	Involved in the biosynthesis of the sialyl Lewis epitope on glycoproteins which in turn have roles in various cellular recognition and communication processes	AR	West Syndrome	Edvardson et al 2013[43]
PIGA	311770	300868	EIEE20	Glycosylphosphatidylinositol (GPI) anchor protein	X-linked	West syndrome<br>(and other EIEE syndromes)<br>multiple congenital anomalies	Belet et al 2013[44]<br>Kato et al 2014[45]
SLC35A2	314375	300896	EIEE22	UDP-galactose transporter – loss of function leads to abnormal galactosylation of glycoproteins	X-linked	Infantile spasms<br>Congenital disorder of glycosylation	Kodera et al 2013[46]<br>Ng et al 2013[47]
DOCK7	615730	615859	EIEE23	Rac guanine nucleotide exchange factor implicated in neurogenesis and the morphological differentiation of GABAergic interneurons in the developing cortex	AR	Infantile spasms<br>Cortical blindness	Perrault et al 2014[48]

Gene	OMIM gene number	OMIM phenotype number	EIEE designation	Gene function	Mode of inheritance	Phenotypes associated with this gene	References
GRIN2B	138252	616139	EIEE27	A subunit of the N-methyl-D Aspartate glutamate receptor with a role in neuronal excitability, and brain development	De novo/AD (gain of function mutations)	Infantile spasms/West syndrome	Lemke et al 2014[49]
SIK1	605705	616341	EIEE30	A member of the AMP kinase subfamily that plays a role in a signal transduction pathway involved in the nuclear regulation of gene expression. Its role in the central nervous system includes regulation of the circadian clock and transcription of corticotropin-releasing hormone in the hypothalamus	De novo	Non- specific EIEE Infantile spasms	Hansen et al 2015[50]
DNM1	602377	616346	EIEE31	GTPase in the pre-synaptic terminal involved in synaptic vesicle endocytosis and membrane recycling, particularly during post-natal development	De novo	Infantile spasms<br>Lennox Gastaut Syndrome	Appenzeller et al 2014[51]
FOXG1	164874	613454	N.A.	A transcriptional repressor. Has a role in differentiation of cortical compartments and neuronal subnuclear localization	De novo/AD	Congenital variant Rett syndrome<br>West syndrome	Striano et al 2011[52]<br>Tohyama et al 2011[53]<br>Brunetti Pierri et al 2011[54]
MEF2C	600662	613443	N.A.	A transcription factor with key roles in both early neuroprogenitor development and neuronal maturation	De novo/AD	Infantile spasms<br>Non- specific EIEE	Novara et al 2010[55]<br>Zweier et al 2010[56]<br>Paciorkowski et al 2013[57]
CHRNA7	118511	604827	N.A.	Encodes alpha-7 subunit of neuronal nicotinic acetylcholine receptors, ligand gated ion channels that mediate synaptic signal transmission	De novo/AD	Generalised epilepsy<br>Infantile spasms	Helbig et al 2009[58]<br>Lacaze et al 2013[59]
CASK	300172	300749	N.A.	A calcium/calmodulin-dependent serine protein kinase which may function as a cytoskeletal membrane scaffold that coordinates signal transduction pathways within the cortical cytoskeleton	De novo/ X linked dominant	Infantile spasms/West syndrome<br>Ohtahara syndrome<br>Early myoclonic epilepsy	Michaud et al 2014[60]<br>Saitsu et al 2012[61]<br>Nakamura et al 2014[62]

Gene	OMIM gene number	OMIM phenotype number	EIEE designation	Gene function	Mode of inheritance	Phenotypes associated with this gene	References
ADSL	608222	103050	N.A.	Enzyme with role in purine synthesis	AR	Non- specific epileptic encephalopathy Infantile	Jurecka et al 2012[63] Maaswinkel-Mooij et al 1997[64] Michaud et al 2014[60]
MAGI2	606382	194050	N.A.	Acts as a scaffold at synaptic junctions, by assembling neurotransmitters and cell adhesion proteins. It may play a role in activin mediated neuronal signaling. Interacts with and stabilizes phosphatase and tensin homolog (PTEN)	De novo/ AD	Infantile spasms Williams Beuren syndrome	Morimoto et al 2003[65] Marshall et al 2008[66]
NF1	613113	162200	N.A.	Encodes neurofibromin, a cytoplasmic protein that is predominantly expressed in neurons, Schwann cells, oligodendrocytes, and leukocytes and has a role in regulating several intracellular processes including tumor suppression	AD	Infantile spasms Neurofibromatosis Type 1	Ruggieri et al 2009[67]
TSC1	605284	191100	N.A.	Encodes hamartin, a protein that interacts with tuberin, to form a complex that inhibits signal transduction to mTOR, a protein kinase which regulates cell growth, proliferation and survival	AD	Infantile spasms/West syndrome Tuberous sclerosis	Hsieh et al 2013[68]
TSC2	191092	613254	N.A.	Encodes tuberin, a protein which interacts with hamartin, to form a complex that inhibits signal transduction to mTOR, a protein kinase which regulates cell growth, proliferation and survival	AD	Infantile spasms/West syndrome Tuberous sclerosis	Hsieh et al 2013[68]
DCX	300121	300067	N.A.	Encodes doublecortin a protein that associates with the microtubule cytoskeleton and has a role in neuronal migration	X-linked	Infantile spasms Lissencephaly- double cortex syndrome	Guerrini et al 2006[69]

Gene	OMIM gene number	OMIM phenotype number	EIEE designation	Gene function	Mode of inheritance	Phenotypes associated with this gene	References
PAFAH1B1	601545	607432	N.A.	Regulates microtubule motor protein and cytoplasmic dynein. Involved in microtubule-dependent cell motility and may stimulate specific dynein functions that are involved in neuronal migration and axon growth	De novo	Lissencephaly 1<br>Infantile spasms	Guerrini et al 2006[69]
PNPO	603287	610090	N.A.	Rate limiting enzyme in Vitamin B6 synthesis.	AR	Pyridoxamine 5'-phosphate oxidase deficiency<br>Non- specific EIEE<br>Infantile spasms/West	Mills et al 2005[70]<br>Mills et al 2014[71]<br>Michaud et al 2014[60]
ALG13	300776	300884	N.A.	Role in catalyzing a key step in Asparagine N-glycosylation, essential to regulation of protein folding and stability	De novo/ X---linked dominant	Non- specific EIEE<br>Infantile spasms/West syndrome	Allen et al 2013[72]<br>Michaud et al 2014[60]
GABRB3	137192	612269	N.A.	Member of the GABA-A receptor gene family of ligand-gated ion channels through which GABA, an inhibitory neurotransmitter acts	De novo	Infantile spasms/West Syndrome<br>Childhood absence epilepsy	Allen et al 2013[72]
KCNJ11	600937	606176	N.A.	Encodes subunit of ATP sensitive potassium channels which couple cell metabolism to membrane excitability in pancreatic beta cells, neurons, endocrine cells, and muscle cells	AD	DEND syndrome<br>Infantile spasms	Bahi-Buisson et al 2007[73]
ATP7A	300011	309400	N.A.	A transmembrane copper-transporting ATPase which functions primarily in the homeostatic maintenance of cell copper levels	X---linked recessive	Menkes Disease<br>Infantile spasms	Prasad et al 2011[74]
AIMP1	603605	260600	N.A.	Role in neurofilament assembly	AR	Hypomyelinating leucodystrophy 3<br>Infantile spasms	Armstrong et al 2013[75]
AKT3	611223	615937*	N.A.	Has a role in signaling cascade which regulates cell proliferation and survival, and maintains balance between catabolism and anabolism	De novo	Megalencephaly-polymicrogyria-polydactyly-hydrocephalus syndrome (MPPH2).<br>Megalencephaly capillary malformation	Nellist et al 2015[76]

Gene	OMIM gene number	OMIM phenotype number	EIEE designation	Gene function	Mode of inheritance	Phenotypes associated with this gene	References
TUBA1A	602529	602529†	N.A.	Important role in microtubule function and stability.	De novo	Lissencephaly 3<br>Multiple brain abnormalities<br>Eye abnormalities (microphthalmia, cataracts)<br>Infantile spasms	Myers et al 2015[77]
KANSL1	612452	610443	N.A.	Member of a histone acetyltransferase (HAT) complex. Has a role in chromatin modification	De novo	Koolen-de Vries syndrome<br>Infantile spasms	Koolen et al 2015[78]
NR2F1	132890	615722‡	N.A.	Has a role in regulation of transcription, important in the development of the eyes and cerebral cortex	De Novo	Bosch-Boonstra-Schaaf optic atrophy syndrome<br>West Syndrome	Hino-Fukuyo et al 2015[79]<br>Michaud et al 2014[60]
BRWD3	300553	300659§	N.A.	Regulates cell morphology and has a role in cytoskeletal organization	X-linked recessive	Mental retardation X-linked 93<br>West Syndrome	Hino- Fukuyo et al 2015[79]
CACNA2D1	114204		N.A.	Encodes the alpha-2/delta subunit of skeletal muscle and brain voltage-dependent calcium channels	De novo	West Syndrome	Hino-Fukuyo et al 2015[79]

EIEE: early infantile epileptic encephalopathy, MPSI: migrating partial seizures of infancy, N.A.: not applicable, AD: autosomal dominant, AR: autosomal recessive, ATP: adenosine triphosphate, DEND: developmental delay, epilepsy, neonatal diabetes.

* Denotes MPPH2, †Denotes Lissencephaly 3, ‡Denotes Bosch-Boonstra-Schaaf optic atrophy syndrome, §Denotes Mental Retardation X-linked 93

References for Supplementary Table 1:

1. Strømme P, Mangelsdorf ME, Scheffer IE, et al. Infantile spasms, dystonia and other X-linked phenotypes caused by mutations in Aristaless related homeobox gene, ARX. *Brain Dev* 2002;24:266-268.

2. Scheffer IE, Wallace RH, Phillips FL, et al. X-linked myoclonic epilepsy with spasticity and intellectual disability: mutation in the homeobox gene ARX. *Neurology* 2002;59:348-356.

3. Guerrini R, Moro F, Kato M, et al. Expansion of the first PolyA tract of ARX causes infantile spasms and status dystonicus. *Neurology* 2007;69:427-433.

4. Kato M, Saitoh S, Kamei A, et al. A longer polyalanine expansion mutation in the ARX gene causes early infantile epileptic encephalopathy with suppression-burst pattern (Ohtahara syndrome). *Am J Hum Genet* 2007;81:361-366.

5. Kato M, Koyama N, Ohta M, et al. Frameshift mutations of the ARX gene in familial Ohtahara syndrome. *Epilepsia* 2010;51:1679-1684.

6. Absoud M, Parr JR, Halliday D, et al. A novel ARX phenotype: rapid neurodegeneration with Ohtahara syndrome and a dyskinetic movement disorder. *Dev Med Child Neurol* 2010;52:305-307.

7. Kalscheuer VM, Tao J, Donnelly A, et al. Disruption of the serine/threonine kinase 9 gene causes severe X-linked infantile spasms and mental retardation. *Am J Hum Genet* 2003;72:1401-1411.

8. Weaving LS, Christodoulou J, Williamson SL, et al. Mutations of CDKL5 cause a severe neurodevelopmental disorder with infantile spasms and mental retardation. *Am J Hum Genet* 2004;75:1079-1093.

9. Scala E, Ariani F, Mari F, et al. CDKL5/STK9 is mutated in Rett syndrome variant with infantile spasms. *J Med Genet* 2005;42:103-107.

10. Bahi-Buisson N, Bienvenu T. CDKL5---Related Disorders: From Clinical Description to Molecular Genetics. *Mol Syndromol* 2012;2:137-152.

11. Fehr S, Wilson M, Downs J, et al. The CDKL5 disorder is an independent clinical entity associated with early-onset encephalopathy. *Eur J Hum Genet* 2013;21:266-273.

12. Molinari F, Raas-Rothschild A, Rio M, et al. Impaired mitochondrial glutamate transport in autosomal recessive neonatal myoclonic epilepsy. *Am J Hum Genet* 2005;76:334-339.

13. Molinari F, Kaminska A, Fiermonte G, et al. Mutations in the mitochondrial glutamate carrier SLC25A22 in neonatal epileptic encephalopathy with suppression bursts. *Clin Genet* 2009;76:188-194.

14. Poduri A, Heinzen EL, Chitsazzadeh V, et al. SLC25A22 is a novel gene for migrating partial seizures in infancy. *Ann Neurol* 2013;74:873-882.

15. Tohyama J, Akasaka N, Osaka H, et al. Early onset West syndrome with cerebral hypomyelination and reduced cerebral white matter. *Brain Dev* 2008;30:349-355.

16. Saitsu H, Kato M, Mizuguchi T, et al. De novo mutations in the gene encoding STXBP1 (MUNC18-1) cause early infantile epileptic encephalopathy. *Nat Genet* 2008;40:782-788.

17. Deprez L, Weckhuysen S, Holmgren P, et al. Clinical spectrum of early-onset epileptic encephalopathies associated with STXBP1 mutations. *Neurology* 2010;75:1159-1165.

18. Otsuka M, Oguni H, Liang JS, et al. STXBP1 mutations cause not only Ohtahara syndrome but also West syndrome- result of Japanese cohort study. *Epilepsia* 2010;51:2449-2452.

19. Saitsu H, Tohyama J, Kumada T, et al. Dominant – negative mutations in alpha II spectrin cause West syndrome with severe cerebral hypomyelination, spastic quadriplegia and developmental delay. *Am J Hum Genet* 2010;86:881-891.

20. Hamdan F, Saitsu, H, Nishiyama K, et al. Identification of a novel in-frame de novo mutation in SPTAN1 in intellectual disability and pontocerebellar atrophy. *Eur J Hum Genet* 2012;20:796-800.

21. Writzl K, Primec ZR, Stražišar BG, et al. Early Onset West Syndrome with severe hypomyelination and coloboma---like optic discs in a girl with SPTAN1 mutation. *Epilepsia* 2012;53:e106-110.

22. Nonoda Y, Saito Y, Nagai S, et al. Progressive diffuse brain atrophy in West syndrome with marked hypomyelination due to SPTAN1 gene mutation. *Brain Dev* 2013;35:280-283.

23. Claes L, Del-Favero J, Ceulemans B, et al. De novo mutations in the sodium- channel gene SCN1A cause severe myoclonic epilepsy of infancy. *Am J Hum Genet* 2001;68:1327-1332.

24. Claes L, Ceulemans B, Audenaert D, et al. De novo SCN1A mutations are a major cause of severe myoclonic epilepsy of infancy. *Hum Mutat* 2003;21:615-621.

25. Escayg A, MacDonald BT, Meisler MH, et al. Mutations of SCN1A, encoding a neuronal sodium channel, in two families with GEFS+2. *Nat Genet* 2000;24:343-345.

26. Mantegazza M, Gambardella A, Rusconi R, et al. Identification of an Nav1.1 sodium channel (SCN1A) loss-of- function mutation associated with familial simple febrile seizures. *Proc Natl Acad Sci U S A*. 2005;102:18177-18182.

27. Dichgans M, Freilinger T, Eckstein, et al. Mutation in the neuronal voltage-gated sodium channel SCN1A in familial hemiplegic migraine. *Lancet* 2005;366:371-374.

28. Freilich ER, Jones JM, Gaillard WD, et al. Novel SCN1A mutation in a proband with malignant migrating partial seizures of infancy. *Arch Neurol* 2011;68:665-671.

29. Carranza Rojo D, Hamiwka L, McMahon JM, et al. De novo SCN1A mutations in migrating partial seizures of infancy. *Neurology* 2011;77:380-383.

30. Wallace RH, Hodgson BL, Grinton BE, et al. Sodium channel alpha1- subunit mutations in severe myoclonic epilepsy of infancy and infantile spasms. *Neurolology* 2003;61:765-769.

31. Dimassi S, Labalme A, Ville D, et al. Whole- exome sequencing improves the diagnosis yield in sporadic infantile spasm syndrome. *Clin Genet* 2015; doi: 10.1111/cge.12636. [Epub ahead of print]

32. Ogiwara I, Ito K, Sawaishi Y, et al. De novo mutations of voltage- gated sodium channel alpha II gene SCN2A in intractable epilepsies. *Neurology* 2009;73:1046-1053.

33. Kamiya K, Kaneda M, Sugawara T, et al. A Nonsense Mutation of the sodium channel gene SCN2A in a patient with intractable epilepsy and mental decline. *J Neurosci* 2004;24:2690-2698.

34. Liao Y, Deprez L, Maljevic S, et al. Molecular correlates of age- dependent seizures in an inherited neonatal-infantile epilepsy. *Brain* 2010;133:1403-1414.

35. Dhamija R, Wirrell E, Falcao G, et al. Novel de novo SCN2A mutation in a child with migrating focal seizures of infancy. *Paediatr Neurol* 2013;49:486-488.

36. Nakamura K, Kato M, Osaka H, et al. Clinical spectrum of SCN2A mutations expanding to Ohtahara syndrome. *Neurology* 2013;81:992-998.

37. Hackenberg A, Baumer A, Sticht H, et al. Infantile epileptic encephalopathy, transient choreoathetotic movements and hypersomnia due to a de novo missense mutation in the SCN2A gene. *Neuropaediatrics* 2014;45:261-264.

38. Martin HC, Kim GE, Pagnamenta AT, et al. Clinical whole- genome sequencing in severe early-onset epilepsy reveals new genes and improves molecular diagnosis. *Hum Mol Genet* 2014;23:3200-3211.

39. Baasch, AL, Hüning I, Gillissen C, et al. Exome sequencing identifies a de novo SCN2A mutation in a patient with intractable seizures, severe intellectual disability, optic atrophy, muscular hypotonia and brain abnormalities. *Epilepsia* 2014;55:e25-29.

40. Kurian MA, Meyer E, Vassallo G, et al. Phospholipase C beta 1 deficiency is associated with early-onset epileptic encephalopathy. *Brain* 2010;133:2964-2970.

41. Poduri A, Chopra SS, Neilan EG, et al. Homozygous PLCB1 deletion associated with malignant migrating partial seizures in infancy. *Epilepsia* 2012;53:e146-150.

42. Ngoh A, McTague A, Wentzensen IM, et al. Severe infantile epileptic encephalopathy due to mutations in PLCB1: expansion of the genotypic and phenotypic disease spectrum. *Dev Med Child Neurol* 2014;56:1124-1128.

43. Edvardson S, Baumann AM, Mühlenhoff M, et al. West Syndrome caused by ST3GAL-III deficiency. *Epilepsia* 2012;54:e24-27.

44. Belet S. Fieremans N, Yuan X, et al. Early Frameshift Mutation in PIGA identified in Large XLID Family without Neonatal Lethality. *Hum Mutat*. 2014;35(3):350-355.

45. Kato M, Saitsu H, Murakami Y, et al. PIGA mutations cause early-onset epileptic encephalopathies and distinctive features**.** *Neurology* 2014; 82(18):1587-1596.

46. Kodera H, Nakamura K, Osaka H, et al. De novo mutations in SLC35A2 encoding a UDP-galactose transporter cause early-onset epileptic encephalopathy. *Hum Mutat.* 2013;34(12):1708-1714.

47. Ng BG, Buckingham KJ, Raymond K, et al. Mosaicism of the UDP-galactose transporter SLC35A2 causes a congenital disorder of glycosylation. *Am J Hum Genet* 2013;92(4):632-636.

48. Perrault I, Hamdan FF, Rio M, et al. Mutations in DOCK7 in individuals with epileptic encephalopathy and cortical blindness. *Am J Hum Genet.* 2014;94(6):891-897.

49. Lemke JR, Hendrickx R, Geider K, et al. GRIN2B mutations in West Syndrome and intellectual disability with focal epilepsy. *Ann Neurol* 2014;75:147-154.

50. Hansen J, Snow C, Tuttle E, et al. De Novo Mutations in SIK1 Cause a Spectrum of Developmental Epilepsies. *Am J Hum Genet*. 2015;96(4):682-690.

51. Appenzeller S, Balling R, Barisic N, et al. De novo mutations in synaptic transmission genes including DNM1 cause epileptic encephalopathies. *Am J Hum Genet.* 2014;95(4):360-370.

52. Striano P, Paravidino R, Sicca F, et al. West syndrome associated with 14q12 duplications harboring FOXG1. *Neurology* 2011;76:1600-1602.

53. Tohyama J, Yamamoto T, Hosoki K, et al. West syndrome associated with mosaic duplication of FOXG1 in patient with maternal uniparental disomy of chromosome 14. *Am J Med Genet A* 2011;155A:2584-2588.

54. Brunetti-Pierri N, Paciorkowski AR, Ciccone R, et al. Duplications of FOXG1 in 14q12 are associated with developmental epilepsy, mental retardation, and severe speech impairment. *Eur J Hum Genet* 2011;19:102-107.

55. Novara F, Beri S, Giorda R, et al. Redefining the phenotype associated with MEF2C haploinsufficiency. *Clin Genet* 2010;78:471-477.

56. Zweier M, Gregor A, Zweier C, et al. Mutations in MEF2C from the 5q14.3q15 microdeletion syndrome region are a frequent cause of severe mental retardation and diminish MECP2 and CDKL5 expression. *Hum Mutat* 2010;31:722-733.

57. Paciorkowski AR, Traylor RN, Rosenfeld JA, et al. MEF2C haploinsufficiency features consistent hyperkinesis, variable epilepsy and has a role in dorsal and ventral neuronal developmental pathways. *Neurogenetics* 2013;14:99-111.

58. Helbig I, Mefford HC, Sharp A, et al. 15q13.3 microdeletions increases risk of idiopathic generalized epilepsy. *Nat Genet* 2009;41:160-162.

59. Lacaze E, Gruchy N, Penniello-Valette MJ, et al. De novo 15q13.3 microdeletion with cryptogenic West syndrome. *Am J Med Genet A* 2013;161:2582-2587.

60. Michaud JL, Lachance M, Hamdan FF, et al. The genetic landscape of infantile spasms. *Hum Mol Genet* 2014;23:4846-4858.

61. Saitsu H, Kato M, Osaka H, et al. CASK aberrations in male patients with Ohtahara syndrome and cerebellar hypoplasia. *Epilepsia* 2012;53:1441-1449.

62. Nakamura K, Nishiyama K, Kodera H, et al. A de novo CASK mutation in pontocerebellar hypoplasia type 3 with early myoclonic epilepsy and tetralogy of Fallot. *Brain Dev* 2014;36:272-273.

63. Jurecka A, Opoka-Winiarska V, Rokicki D, et al. Neurologic presentation, diagnostics, and therapeutic insights in a severe case of adenylosuccinate lyase deficiency. *J Child Neurol* 2012;27:645-649.

64. Maaswinkel-Mooij PD, Laan LA, Onkenhout W, et al. Adenylosuccinase deficiency presenting with epilepsy in early infancy. *J Inherit Metab Dis* 1997;20:606-607.

65. Morimoto M, An B, Ogami A, et al. Infantile spasms in a patient with Williams syndrome and craniosynostosis. *Epilepsia* 2003;44:1459-1627.

66. Marshall CR, Young EJ, Pani AM, et al. Infantile spasms is associated with deletion of the MAGI2 gene on chromosome 7q11.23---q21.11**.** *Am J Hum Genet* 2008;83:106-111.

67. Ruggieri M, Iannetti P, Clementi M, et al. Neurofibromatosis type 1 and infantile spasms. *Childs Nerv Syst* 2009;25:211-216.

68. Hsieh DT, Jennesson MM, Thiele EA. Epileptic spasms in tuberous sclerosis complex. *Epilepsy Res* 2013;106:200-210.

69. Guerrini R, Marini C. Genetic malformations of cortical development. *Exp Brain Res* 2006;173:322-333.

70. Mills PB, Surtees RA, Champion MP, et al. Neonatal epileptic encephalopathy caused by mutations in the PNPO gene encoding pyridox(am)ine 5'-phosphate oxidase. *Hum Mol Genet* 2005;14:1077-1086.

71. Mills PB, Camuzeaux SS, Footitt EJ, et al. Epilepsy due to PNPO mutations: genotype, environment and treatment affect presentation and outcome. *Brain* 2014;137:1350-1360.

72. Allen AS, Berkovic SF, Cossette P, et al. De novo mutations in epileptic encephalopathies. *Nature* 2013;501:217-221.

73. Bahi-Buisson N, Eisermann M, Nivot S, et al. Infantile spasms as an epileptic feature of DEND syndrome associated with an activating mutation in the potassium adenosine triphosphate (ATP) channel, Kir6.2. *J Child Neurol* 2007;22:1147-1150.

74. Prasad AN, Levin S, Rupar CA, et al. Menkes disease and infantile epilepsy. *Brain Dev* 2011;33:866-876.

75. Armstrong L, Biancheri R, Shyr C, et al. AIMP1 deficiency presents as a cortical neurodegenerative disease with infantile onset. *Neurogenetics* 2014;15(3):157-159.

76. Nellist M, Schot R, Hoogeveen-Westerveld M, et al. Germline activating AKT3 mutation associated with megalencephaly, polymicrogyria, epilepsy and hypoglycemia. *Mol Genet Metab.* 2015;114(3):467-473.

77. Myers KA, Bello-Espinosa LE, Kherani A, et al. TUBA1A Mutation Associated with Eye Abnormalities in Addition to Brain Malformation. *Pediatr Neurol*. 2015; doi:10.1016/j.pediatrneurol.2015.07.004. [Epub ahead of print]

78. Koolen DA, Pfundt R, Linda K, et al. The Koolen-de Vries syndrome: a phenotypic comparison of patients with a 17q21.31 microdeletion versus a KANSL1 sequence variant. *Eur J Hum Genet.* 2015; doi: 10.1038/ejhg.2015.178. [Epub ahead of print]

79. Hino-Fukuyo N, Kikuchi A, Arai-Ichinoi N, et al. Genomic analysis identifies candidate pathogenic variants in 9 of 18 patients with unexplained West syndrome. *Hum Genet*. 2015; 134(6):649- 658.
